# Application of New Materials in Auditory Disease Treatment

**DOI:** 10.3389/fncel.2021.831591

**Published:** 2022-01-31

**Authors:** Ming Li, Yurong Mu, Hua Cai, Han Wu, Yanyan Ding

**Affiliations:** Department of Otorhinolaryngology, Union Hospital, Tongji Medical College, Huazhong University of Science and Technology, Wuhan, China

**Keywords:** new materials, auditory diseases, conductive hearing loss, sensorineural hearing loss, therapy

## Abstract

Auditory diseases are disabling public health problems that afflict a significant number of people worldwide, and they remain largely incurable until now. Driven by continuous innovation in the fields of chemistry, physics, and materials science, novel materials that can be applied to hearing diseases are constantly emerging. In contrast to conventional materials, new materials are easily accessible, inexpensive, non-invasive, with better acoustic therapy effects and weaker immune rejection after implantation. When new materials are used to treat auditory diseases, the wound healing, infection prevention, disease recurrence, hair cell regeneration, functional recovery, and other aspects have been significantly improved. Despite these advances, clinical success has been limited, largely due to issues regarding a lack of effectiveness and safety. With ever-developing scientific research, more novel materials will be facilitated into clinical use in the future.

## Introduction

According to the latest World Health Organization estimates, 466 million people around the world (over 5% of the world population) experience disabling hearing loss. By 2050, this number is projected to rise to approximately 900 million, and nearly 2.5 billion people are at risk of contracting auditory diseases (World Health Organization [WHO], 2021). The early detection, vaccination, accurate management, and timely treatment of auditory diseases can help improve clinical outcome. Nevertheless, therapeutic modalities and prevention strategies for the occurrence and development of auditory diseases are still limited currently. Hearing impairment caused by auditory diseases is categorized into three clinical types: conductive (CHL), sensorineural (SNHL), and mixed (MHL) hearing loss. CHL is known to result primarily from structural damage, blockage, and sclerosis of the outer and middle ear, eventually leading to aberrant signaling to the inner ear. Disruption of the inner ear, auditory nerve, central auditory nuclei, or cortex are classified as SNHL, with an elaborate pathology that includes loss of sensory hair cells, spiral ganglion neurons (SGNs), and stria vascularis cells in the inner ear, ultimately leading to the failure of auditory perception (Ma et al., 2019). The established therapy for patients suffering from conductive deafness focuses on middle-ear infection, otosclerosis, etc. Research on curative therapies for sensorineural hearing loss mainly focuses on the repair and regeneration of hair cells, stria vascularis, and nerve synapses.

**FIGURE 1:**
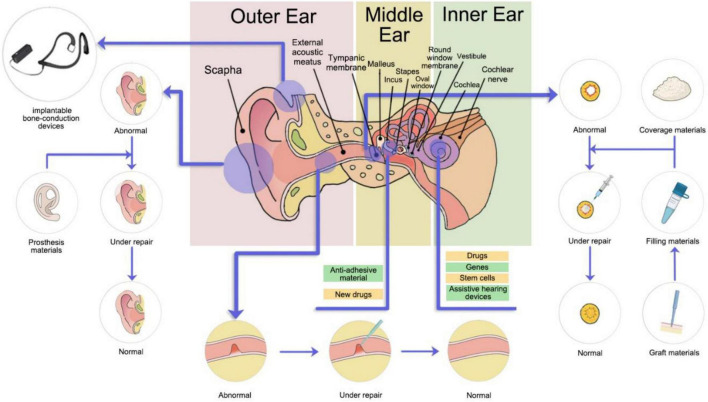
Treatment sites of new materials for auditory diseases.

The practical application of new materials is continuously undergoing considerable advancements, and substantial success has been achieved in some aspects. Whereas the new materials are being applied to treat auditory diseases, the abundant advances made in the fields of diabetes, cardiovascular disease, and neuromuscular disease using new materials are progressing. Recently, the Food and Drug Administration (FDA) approved the first RNA interference-based gene silencing technology drug—Patisiran—which regulates gene expression by the delivery of RNA to target cells, improving the prognosis of patients with rare cardiac and neurologic disease (Adams et al., 2018). For another example, with a fundamental role in the future repair or replacement of tissues defects (Zakrzewski et al., 2019), stem cells differentiate into insulin-producing cells after being implanted into the body, bringing considerable improvements to the prognosis of type 1 diabetes (Shahjalal et al., 2018). Regarding to stem cell therapy, graphene, with remarkable biocompatible and bioadhesive properties, can be fabricated as scaffolds for the proliferation and direct differentiation of stem cells (Kenry Lee et al., 2018). Some scholars even propose stem cell engraftment as a highly feasible and fundamental curing method for sensorineural deafness (Nakagawa and Ito, 2005; Cheng et al., 2019). Many prostheses formed out of new materials were reported to induce fewer immune responses and had a better overall prognosis than conventional materials after implantation (Diken Turksayar et al., 2019; Nappi et al., 2021). This review will focus on the research progress of the novel materials employed for the treatment of CHL and SNHL (see Figure 1).

## Treatment Orientation of Hearing Loss

Hearing loss is attributable to genetic factors, specific viral infections, chronic ear infections, birth complications, exposure to excessive noise, aging, and ototoxic drugs. CHL may occur as middle-ear effusion, tympanic membrane perforation, physical external trauma, infection, canal stenosis, cholesteatoma, otitis media, otosclerosis, ossicular erosions, and so on (Ontario Health, 2020). At present, treatment for CHL primarily involves surgery and drugs. Primary research directions for novel therapies are focused on new pharmaceuticals, materials, artificial auditory implantation, and other aspects. The artificial auditory implantation comprises middle-ear implant (MEI) and implantable bone-conduction devices (Chen et al., 2014). The current widespread adoption of artificial auditory implantation includes bone-anchored hearing aids (BAHAs), subcutaneous bone bridges (BBs), vibrant sound bridges (VSBs), and semi-implantable middle-ear transducers. However, due to the pathway for bone conduction being more complex than air conduction, the following areas must be considered: percutaneous attenuation of high-frequency sound after the implantation of bone-conduction hearing aid devices; the susceptibility to infection of surgical sites after BAHA implantation; vibrator displacement after MEI implantation; ossicular necrosis; the wide fluctuation of postoperative gain, and so on. Therefore, further research aimed at bone-conduction audiology and more development of audiological assist devices are still required (Ghoncheh et al., 2016).

Diseases that could contribute to SNHL are age-related hearing loss (ARHL), inherited hearing impairments, Meniere’s disease (endolymphatic hydrops), autoimmune inner-ear disease, ear infection, drug-induced deafness, ear trauma, and idiopathic SNHL (Merchant and Nadol, 2001; Kanzaki et al., 2020). In addressing sudden sensorineural hearing loss (SSNHL), current SSNHL management guidelines recommend glucocorticoids, psychotherapy, and intravenous agents that can improve microcirculation and neurotrophy, besides which numerous attempts for novel therapies have been reported. For instance, Vanwijck et al. (2019) summarized the efficacy of intratympanic injections of corticosteroids with a Silverstein tube to treat refractory SSNHL. They found that the topical application of corticosteroids with such a tube to the inner ear through the round window membrane can improve the hearing and clarity of patients who have failed in previous conventional therapies. However, therapies for SNHL remain clinically limited thus far, and the therapeutic effects remain less than satisfactory. Owing to the natural anatomical and physiological barriers of the cochlea, the supply and absorption of drugs reaching the targeting cochlea cells have been severely hindered (Zou et al., 2016; Yuan and Qi, 2018). An example is that the cochlea, surrounded by bones and located in a relatively closed environment, is difficult for drugs to access from the blood due to the presence of the blood labyrinth barrier (BLB). For local drug delivery to the cochlea, drug penetration through the oval window (OW) and round window membrane (RWM) becomes difficult due to their permselectivity. In addition, some of the drugs have a short half-life, and inter-individual differences in metabolism are marked, therefore limiting the benefits of drugs administered using traditional approaches.

In recent years, a range of new signaling pathways and genes playing essential roles in hair cell development and differentiation have been discovered that target therapies against SNHL (Diensthuber and Stover, 2018). For example, overexpressing Atoh1 can potentially reprogram supporting cells to become hair cells, subsequently promoting mammalian hair cell regeneration (Richardson and Atkinson, 2015). In cell cycle regulation, the absence of p27Kip1 drives the enhanced proliferation of supporting cells in adult mice, and inactivation of the retinoblastoma 1 (RB1) gene leads to the acquisition of newly generated hair cells originated from highly differentiated hair cells (Sage et al., 2005). For other signaling pathways, the activator of the Wnt signal pathway—β-catenin, the overexpression of which triggers Lgr5-positive cell growth and mitosis—elevates hair cell regeneration (Shi et al., 2013). When β-catenin and Atoh1 are co-expressed, the differentiation of Lg5-positive hair cells in neonatal mice is significantly advanced (Kuo et al., 2015). Furthermore, inhibition of the Notch pathway *via* γ-secretase inhibitors enables mice to regenerate hair cells in response to noise-induced damage, simultaneously gaining an improvement in their hearing level (Mizutari et al., 2013). Furthermore, there is crosstalk between Notch and Wnt signaling (Li et al., 2015; Ding et al., 2020). Hence, various predictions can be hypothesized based on these theories; that is, gene targets that can be selectively modulated using small molecules or related drugs might drive the regeneration of hair cells.

## Application of Novel Materials in Hearing Loss

### Treatment of New Materials in Conductive Hearing Loss

In auricle-related disorders, the microtia is a congenital craniofacial malformation, ranking only second to cleft lip (Jiao and Zhang, 2001). Traditional healing strategies employ autologous cartilage as conventional grafts (Bichara et al., 2012). Shang et al. (2016) used 3D printing technology and medical silicone to fabricate an auricular scaffold, and they found the scaffolds were implanted *in vivo* smoothly, with excellent bio-compatibility, affordable cost, and a simple preparation process. Presently, besides adipose-derived stem cell (ADSC) application for cartilage regeneration, cartilage stem/progenitor cells (CSPCs) have also been used for the successful ear reconstruction and modeling the pathogenesis of auricular deformities (Zhou G. et al., 2018; Zucchelli et al., 2020).

In tympanic-associated disorders, tympanic membrane perforation (TMP) is a commonly seen disease in otorhinolaryngology. Although the preferred autograft materials, such as temporal muscle, cartilage, and fat, have a variety of applications, they have also been reported to be highly susceptible to infection and have pronounced dependence on nutrients from the surrounding tissue (Teh et al., 2013). Therefore, an increasing emphasis is being placed on new materials that can decrease the rate of inflammation, foster tympanic membrane regeneration, and enhance the proliferation and migration of the epithelium. The current methods for tympanic membrane repair combine autologous tissue and novel materials, the latter including platelet-rich plasma (PRP), platelet-rich fibrin (PRF), hyaluronic acid (HA), epidermal growth factor (EGF), and basic fibroblast growth factor (bFGF). Among them, PRP and PRF have gained particular interest because they are enriched in growth factors, which are effective in inducing tissue regeneration and with a lower prevalence of infections (Gur et al., 2016; Stavrakas et al., 2016; Huang et al., 2021). Moreover, a study by Lin et al. (2013) found that recombinant human epidermal growth factor (rhEGF) in conjunction with a gelatin-based sponge scaffold can remarkably enhance the wound healing rate of chronic TMP, reduce improvement time, further improve hearing level, and has no toxic side effects on inner-ear function. Similar multiple studies have been performed for combining new materials into clinical practice, and correspondingly, an increased likelihood of TMP healing has been reported (Gisselsson-Solen et al., 2020). For example, heparin-binding epidermal growth factor (EGF)-like growth factor (Santa Maria et al., 2015), EGF-released nanofibrous patches (Seonwoo et al., 2019b), bacterial cellulose (Mandour et al., 2019), chitosan patch scaffolds incorporated with insulin-like growth factor-binding protein 2 (Seonwoo et al., 2019a), and a fibroblast growth factor (FGF)-infiltrated gelatin sponge (Omae et al., 2017; Kanemaru et al., 2018) have been documented to accelerate tympanic membrane (TM) perforation healing. As a type of novel material, Maharajan et al. (2020) have also reported that mesenchymal stem cells (MSCs) are promising candidates for therapy to treat TMP. Either separate engraftment of multipotent MSCs or a combination of these cells with biological materials and growth factors (GFs) can achieve faster TMP healing *via* increasing the activation of epidermal stem cell markers and stimulating the proliferation and migration of keratinocytes. MSCs, scaffolds, and GFs have a synergistic role in TM regeneration. The recent advancement of 3D- and 4D-printing technology has driven the development of a precise MSC-attached scaffold designed for the physical structure of patients. The direction of physical and chemical engineering may be incorporated in this advancement, further boosting the degree and rate of the target tissue regeneration (Maharajan et al., 2020). The introduction of a cobalt-chromium (Co-Cr) coronary stent into the eustachian tube potentiates middle-ear ventilation, as validated in sheep (Pohl et al., 2018). A new class of tympanostomy stent made of nickel and titanium with a TiO2 coating has previously been shown to reduce *Pseudomonas aeruginosa* biofilm formation, resulting in a lower incidence of postoperative complications, such as deafness and catheter blockage after tympanotomy tube insertion (Joe and Seo, 2018).

Tympanoplasty exerts significant actions on the treatment of auditory diseases such as chronic otitis mastoidea, tympanosclerosis, cholesteatomatous chronic otitis media, and middle-ear sound transmission defect caused by trauma. Adhesive otitis patients often require surgical interventions when they present with concomitant middle-ear atelectasis, structural adhesion in the tympanic chamber, sound transmission disorder, recurrent infection, and persistent otorrhea (Larem et al., 2016). To avoid postoperative adhesion and to clean effusion, the filler materials are required for adhesion prevention after removal of tympanic sclerotic foci and middle-ear blockage caused by pathological factors; these materials can be divided into two categories: absorbable and non-absorbable. The most recent anti-adhesive materials include chitosan hydrogel within absorbable materials (Unsaler et al., 2016) and natural polymeric biomaterials–Naso Pore (Huang et al., 2011). These two ingredients are otherwise used for nasal surgery and are of low ototoxicity, inflammatory response, and have strong anti-adhesion effects (Chen and Li, 2020). In the medical management of middle-ear cholesteatoma, traditional surgery combined with novel materials has been found to have superior efficacy. For mastoid postoperative tamponade, synthetic materials have clear advantages of easy accessibility, short procedure time, being less prone to contamination and so on, compared with traditional autologous materials, which are less accessible and unstable. Common filling synthetic materials in mastoid disorders include bioactive glass, hydroxyapatite, titanium, and silicon, with bioactive glass being most frequently used (Lee H. B. et al., 2013; Sorour et al., 2018). Tympanoplasty necessitates the use of transplanted materials, but classically employed autogenous materials are with high risk of shift, complete absorption, and residual cholesteatoma. Bartel et al. (2018) determined that total (TORP) and partial (PORP) ossicular replacement prostheses can be biosynthesized and are stiff, safe, and stable. Compared with traditional materials, the hearing improvement after ossicular chain reconstruction is almost equivalent to using biosynthesizing materials, and the curative effect of myringoplasty is even superior to that of traditional autologous grafting materials (Bartel et al., 2018; Li et al., 2021).

The ear is one of the predilection sites for keloids, frequently following trauma, surgery, burns, and ear piercing. Due to the high rate of recurrence, the search for excellent postoperative coverage materials and preventing recurrence has become a particular research hotspot in the treatment of keloids (Du and Zhu, 2015). Park et al. proposed that employing hydrocolloid dressing as a wound coverage and pressurizing with a magnet during the early postoperative period, compared with traditional dressings used to cover wounds, could protect the wound tissue, facilitate the healing process, and reduce the water content inside the wound tissue (Park and Chang, 2013). Besides, more coverage materials to promote wound healing and reduce inflammation will become available in the coming years. For patients with CHL but normal hearing function of the inner ear, other assistive hearing devices are increasingly promising in addition to the aforementioned BAHA, BB, vibrant sound-bridge (VSB), and middle-ear transducers, such as ADHEAR–a new non-invasive bone-conduction hearing-assistive device, which uses a cohesive adaptor affixed to the skin surface behind the ear and applies no pressure to the skin (Brill et al., 2019).

### Treatment of New Materials in Sensorineural Hearing Loss

The loss of outer hair cells and spiral ganglion cell degeneration are major causes of sensorineural hearing loss (Wong and Ryan, 2015). The loss of hair cells, spiral ganglion cells, and auditory nerve fibers in the adult ear is irreversible, leading to permanent SNHL. The surgical implantation of a cochlear implant (CI) is envisaged as one of means to restore hearing, but the function of artificial cochlea is highly reliant on residue numbers of spiral ganglion cells. A recent study has found that superparamagnetic iron oxide (SPIO) nanoparticles can direct spiral ganglion neurites to orient to a CI electrode under the application of magnetic field modulation and maintain the survival of SGNs, producing a positive CI treatment effect (Hu et al., 2021). Various other *in vitro* experiments have newly demonstrated that the cGMP-dependent atrial natriuretic peptide (ANP) and the permissive environment created by novel silicon micro-pillar substrates (MPS) could facilitate the survival and growth of SGNs (Mattotti et al., 2015; Sun et al., 2020). Through *in vivo* experiments, mesenchymal stem cells (Maharajan et al., 2021), valproic acid (VPA; 2-propylpentanoic acid) with growth factors (Wakizono et al., 2021), as well as neural stem cells (He et al., 2021) have been found to be beneficial for SGN growth.

**FIGURE 2:**
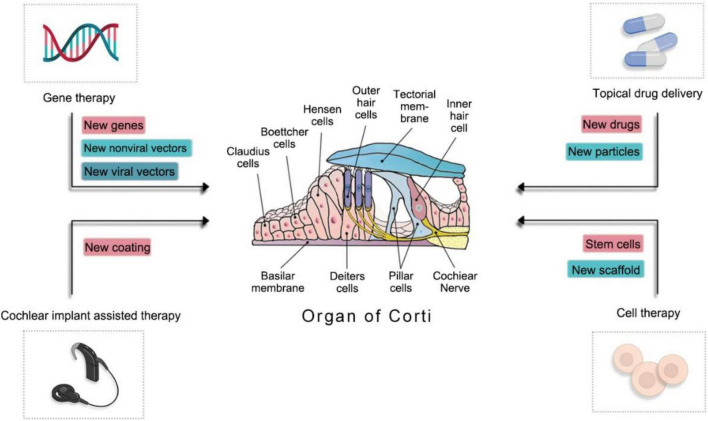
The mechanism of novel materials involved in inner-ear regeneration.

Additionally, to achieve more SGNs and hair cell regeneration, researchers have been studying drug delivery systems (DDSs), gene therapy, and cell therapy from different perspectives (Ma et al., 2019) (see Figure 2).

The clinical advancement of DDS has been hindered by a short biological half-life, poor pharmacokinetics, and low permeability through the biological barriers of nerve growth factor (NGF). Hence, developing highly efficient and inexpensive materials is of substantial importance (Bartus, 2012; Khalin et al., 2015). Currently, the nanotechnology-based ongoing development of novel NGF delivery systems covers nanogels, hydrogels, micelles, microspheres, electrospun nanofibers, nanoparticles, and supraparticles. NGF could be immobilized in nanomaterials *via* physical trapping, adsorption, or electrostatic interactions, then the sustained release can be obtained using diffusion of NGF and/or degradation of carriers, with the aim of local, sustained drug release (Ma et al., 2019). One such nanoscaled drug delivery system could be used in targeted drug delivery. By constructing degradable and non-toxic nanoparticle loading drugs, ligands for the specific recognition and delivery into targeted organs or tissues, nanoscaled drug delivery systems show the stability and property of sustained release (Ding et al., 2019; An and Zha, 2020). Moreover, supraparticles have increased volume in comparison to nano-onions, which have yet to be fully translated into clinical practice. The supraparticles have a larger capacity for the delivery of drugs and provide sustained drug release, concurrently spreading to particular targeted areas (Ma et al., 2018). However, several challenges persist for the clinical application of nanoparticle drug delivery systems, such as drug delivery risk assessments through the RWM or OW routes and the implementation of controlled release and targeted nano-drug delivery. For example, intratympanic medication injection or nanoparticle delivery may access the inner ear more through the RWM approach (Du et al., 2013; Liu et al., 2013), but OW shows higher permeability than RWM (King et al., 2011, 2013; Zou et al., 2012). Chen et al. also found similar results in that fluorescence traceable chitosan nanoparticles (CS-NPs) were more quickly transported into the inner ear through OW, with less damage exhibited (Ding et al., 2019), but further validation regarding nanomaterials and loaded drugs for optimum routes of delivery is still necessary. In gene therapy, the introgressed gene in the inner ear typically involves the basic helix-loop-helix (bHLH) family transcription factor (Shou et al., 2003) as well as neurotrophic (Sun et al., 2011), anti-apoptotic (Chan et al., 2007; Pfannenstiel et al., 2009), antioxidant (Kawamoto et al., 2004), connexin (Birkenhager et al., 2006; Sun et al., 2009), cochlear protein-related, interleukin, and other (Zhuo et al., 2008) genes. To cross the BBB and blood labyrinth barrier successfully and transfer functional genes into the mammalian inner ear, both viral and non-viral vectors are the most common tools for transgene delivery. Viral vectors consist of adenovirus (AV), adeno-associated virus (AAV), herpes simplex virus type I, vaccinia virus, and so on. Non-viral vectors can be categorized into liposomes, cationic polymers, peptide-based nanoparticles, and other synthetic vectors (Sun and Wu, 2013). Other vectors, such as viral and non-viral composite carriers, as well as bacterial vectors, are being studied (Akin et al., 2007). AAV2/Anc80L65, a recently discovered synthetic viral vector, targets the inner and outer hair cells of the cochlea with high efficiency and elicits only a weak immune response when compared to other viral vectors with low transfection ability (Zinn et al., 2015). Tan et al. designed a variant of AAV—AAV-inner ear (AAV-ie). The variant not only achieved 90% successful transfection to supporting cells but also expanded the number of mice hair cells *via* carrying the Atoh1 gene, which did not affect the numbers of hair cells or hearing level (Tan et al., 2019). MicroRNA regulates gene expression *in vivo* by means of inhibiting protein translation and promoting mRNA degradation, the primary function of which is to play a suppressor role post-transcriptionally (Wu et al., 2020). Luo et al. constructed MicroRNA-146a (miR-146a) lentiviral recombinant vectors and found that miR-146a could significantly mitigate inflammatory injury and auditory dysfunctions of the inner ear after being injected into the inner ear through the scala tympani (Luo et al., 2016). A single microRNA can regulate hundreds of different transcription processes, by which the regeneration of damaged hair cells in the inner ear and treating hearing loss are major focuses of much recent research (Zhou W. et al., 2018). Autoimmune inner ear disease (AIED) is an independent disorder, the main clinical manifestation of which is bilateral, rapidly progressive sensorineural hearing loss. Distinct from conventional systemic glucocorticoid treatment, Cai et al. injected adenoviral-mediated interleukin-10 (IL-10) through the RWM. The authors found that adenoviral carrying IL-10 can be imported into the inner ear of guinea pigs and express the gene products, consequently attenuating inflammatory injury and impairment in the auditory function of the inner ear to some extent (Cai and Tan, 2015). Therapies for sensorineural hearing loss by replacement of damaged or lost cells and secreting neurotrophic factors, cytokines, immunoregulatory protein, etc., with the transplanted cells hold promise in a wide variety of fields (Mehrabani et al., 2016; Kanzaki, 2018). Mesenchymal (MSCs), embryonic (ESCs), and induced pluripotent (iPSCs) stem cells are the most frequently used sources for regenerative medicine in the field of otology. Nevertheless, in contrast, the potential tumorigenicity and immune rejection response caused by ESCs and iPSCs, MSCs have a higher safety profile, anti-apoptotic properties, and characteristics of easy amplification; hence, MSCs have become a particular focus of regenerative medicine research (Kaboodkhani et al., 2021). The application of MSCs promotes BDNF secretion and increases the expression of connexin26 and connexin30, contributing to the alleviation of inflammation, oxidative stress, and apoptosis, the recovery of cell-cell junctions, an increased number of SGNs differentiated into neural progenitor cells and specific lineages of neurons, and significantly decreased hearing threshold and improved audial function (Matsuoka et al., 2007; Jang et al., 2015; Young et al., 2018; Mittal et al., 2020). In regenerative medicine, scaffolds are essential for cell growth and could provide a three-dimensional structure for cell differentiation and migration to restore the normal function of damaged organs (Khodakaram-Tafti et al., 2017). The preparation of scaffolds can be accomplished with various source materials, including decellularizing tissue (Hashemi et al., 2018), 3D printing technologies (Wang and Yang, 2021), tailored hydrogels (Slaughter et al., 2009), and electrospinning (Malik et al., 2021). Recent studies have used biological scaffolds comprising 3D graphene and artificial photonic crystals for stem cell culture. Scaffolds mimicking the microenvironment of cell growth *in vivo* can positively regulate stem cell growth, proliferation, and differentiation (Yang et al., 2013; Ankam et al., 2015; Fang et al., 2019). Other novel materials yielding similarly supportive effects for stem cells include 3D polydopamine-functionalized carbon microfibrous scaffolds (Yang et al., 2020), Ti_3_C_2_T_x_Mxene membranes (Guo et al., 2020), microfluidic chip platforms (Park et al., 2014), and anisotropy inverse opals (Pettinato et al., 2015). Suggested mechanisms of the aforementioned materials include the promotion of cell growth, expansion, development and cell-cell adhesion, alignment, and connection.

In terms of hearing ancillary equipment, the application of CI for bilateral severe-to-profound SNHL has also reached maturity. However, implantation surgery can potentially cause invasive damage. Recent studies have increasingly found that degradable cell coatings for CI electrodes can increase neuronal survival by persistently releasing BDNF from cell-coated surfaces (Richardson et al., 2009). In addition, the local delivery of dexamethasone into the inner ear or coating *Cis* with dexamethasone during cochlear implantation may alleviate inflammation in the surgical sites (Farhadi et al., 2013; Lee J. et al., 2013).

## Other Novel Materials for Treating Auditory-Related Conditions

The control of tinnitus by external noise stimuli has become a therapy of particular interest. Tinnitus afflicts approximately 15% of the general population worldwide, and there is currently no cure for this disorder. Attempts have been made to ameliorate tinnitus with individually applied spectrally optimized near-threshold noise (Schilling et al., 2021), with most patients stating that subjective tinnitus loudness was suppressed during the noise stimulation.

The discovery of novel pharmaceuticals is one of the important directions for future research. Today, drug candidates with potential therapeutic applications for hearing loss include antioxidants, regulators of mitochondrial function, ion channel modulators, hair-cell protectants, and anti-inflammatory drugs (Ji et al., 2021). Other potentially effective drugs that have been experimentally validated are N-acetylcysteine (NAC) (Marie et al., 2018), melatonin (Serra et al., 2020), resveratrol (Xiong et al., 2015), mineralocorticoid aldosterone (ALD) (Campos-Banales et al., 2015; Halonen et al., 2016), teprenone (GGA) (Mikuriya et al., 2008), aspirin (Verschuur et al., 2014), and salicylic acid (Li et al., 2020) et al. One example is age-related hearing loss (ARHL); SAMP8 mice models demonstrated notably decreased auditory brainstem response (ABR) thresholds, increased distortion product otoacoustic emission (DPOAE) amplitudes, and memory improvement after the administration of NAC, suggesting that NAC has protective effects on hair cells and neurons of the central nervous system (CNS) (Li et al., 2020). Some systemic diseases are accompanied by damage to the inner ear, such as the microvascular and nerve damage caused by long-term diabetes, further leading to neurovascular injury in the cochlear. Gao et al. (2018) found resveratrol to be effective in preventing cell apoptosis and reducing diabetes damage to the inner ear by hypoglycemic activities, anti-dyslipidemic effects, and inhibiting anti-oxidative stress, ICAM-1, and VEGF expression. GGA was found to up-regulate heat-shock proteins (HSPs), and many experimental studies have demonstrated the protective effect of HSPs against cochlear damage induced by noise exposure, ototoxic drugs, and heat stress. In addition, a decline of ABR thresholds was observed in presbycusis mice (Mikuriya et al., 2008). ARHL models of DBA/2J mice were given injections of 200 mg/kg salicylic acid, and the expression of prestin, hair-cell survival, and function of the cochlea were significantly better than in saline-treated controls (Li et al., 2020). However, although antioxidants, such as resveratrol, ferulic acid (Fetoni et al., 2010), and glutathione (Ohinata et al., 2000) are currently under active research, Sha Suhua et al. expressed caution that long-term or excessive intake of exogenous antioxidants or vitamin supplements is not only hazardous to hearing but also increases the risk of developing cancer (Sha and Schacht, 2017). Additionally, Kashio et al. (2009) and Polanski et al. identified that various antioxidants such as Ginkgo biloba extract (GBE), alpha-lipoic acid (AL), and vitamins A, E, and C do not result in hearing improvement (Polanski and Cruz, 2013). For other novel agents, bioactive peptides from Rapana venosa were found to exert an antioxidant effect, as demonstrated *via* the diminishing hair-cell uptake of gentamicin, activating Nrf2-Kcap1-ARE, up-regulation of Nrf2, and the expression of related antioxidant genes without damaging mechanoelectrical transduction channels (Yan, 2021). It has been suggested that rapamycin can slow the onset of ARHL through the inhibition of mTORC1 hyperactivation (Fu et al., 2018). Multiple previous studies have also shown that rapamycin reduces cisplatin- and gentamicin-induced nephrotoxicity (Fang and Xiao, 2014; Ebnoether et al., 2017) and alleviates noise-induced hearing loss (NIHL) by reducing oxidative stress (Yuan et al., 2015). However, rapamycin has many side effects as an immunosuppressant. New drugs can simulate the antioxidant activity of rapamycin with fewer side effects, and such classes of drugs are currently undergoing clinical experimentation. Traditional herbal medicines have been used in China for thousands of years. However, to date, the efficacy of these medicines has yet to be confirmed, and their action mechanisms remain unclear. Modern pharmacological studies have also spurred intense research programs targeting the auditory diseases. For instance, Xuan et al. (2018) demonstrated that composite Jianer formulations, mainly consisting of Puerariae Lobatae Radix (Gegen in Chinese), Scutellariae Radix (Huangqin in Chinese), and Salvia miltiorrhiza (Danshen in Chinese), reduced the level of malondialdehyde (MDA), the main product of lipid peroxidation, to exhibit an antioxidant effect in adult mice. Besides, the composite Jianer formulations ameliorated mtDNA damage and the release of Cyt-c, thereby attenuating caspase-mediated apoptosis through the mitochondrial pathway and playing a protective role against age-related decline in hair cells and spiral ganglion neurons. Furthermore, traditional Chinese herbal medicine (TCHM) seems to be associated with few adverse effects (Xuan et al., 2018). Another active ingredient extracted from traditional Chinese medicine Flos puerariae (Gehua in Chinese)—total flavonoids of Pueraria lobata—has been found to suppress the inflammatory response. The total flavonoids of Pueraria lobata reduce isoprenaline-induced damage to the inner ear of the rat *via* inhibiting the expression of TNF-α, IL-4, and Bax and up-regulating ACTH proteins, at the same time regulating auditory system homeostasis and improving the microvasculature circulation of the inner ear and the ischemia conditions of tissue cells in rats (Zhao and Liang, 2018). Thus, TCHM has substantial potential for treating auditory diseases. The development of new classes of novel antibiotics is valuable for the treatment of hearing loss. For example, for severe chronic suppurative otitis media, new antibiotics are required for effective anti-infectious therapeutics owing to the constant emergence of drug-resistant bacteria. A kind of new cephalosporin— “ceftolozane-tazobactam”—has particularly good therapeutic benefits for moderate to severe suppurative otitis media caused by multidrug- and extensively drug-resistant (MDR and XDR) *P. aeruginosa* (Saraca et al., 2019).

This study has highlighted some bottlenecks in the application of therapeutic materials for auditory diseases, such as the tumorigenicity of repair-promoting substances, immune reaction to implanted materials, high cost, and a difficult fabrication process. More research combining materials science, basic medicine, and clinical medicine is required to address these bottlenecks, with the aim of obtaining more cost-effective and safer new materials for treating hearing loss.

## Conclusion

Presently, various deficiencies still exist regarding insufficient treatment options and their modest efficacy for auditory diseases, leading to CHL and SNHL. Nevertheless, the development and application of novel materials are gradually improving the condition. The novel materials are able to hasten perforated tympanic membrane healing, tympanoplasty of the middle ear, and repair of the microtia, as well as promote the regeneration of inner hair cells, spiral ganglion cells, and the synaptic ribbons, decrease inflammation level, and modify hearing level adjunctively. Although the efficacy and safety of novel materials in clinical application remain to be verified, with more progression of research and substantial technological innovation, these novel materials will be continuously improved, and more refined materials could be employed to treat hearing loss clinically.

## Author Contributions

ML conceived and wrote the manuscript. YD modified the manuscript. All authors contributed to the article and approved the submitted version.

## Conflict of Interest

The authors declare that the research was conducted in the absence of any commercial or financial relationships that could be construed as a potential conflict of interest.

## Publisher’s Note

All claims expressed in this article are solely those of the authors and do not necessarily represent those of their affiliated organizations, or those of the publisher, the editors and the reviewers. Any product that may be evaluated in this article, or claim that may be made by its manufacturer, is not guaranteed or endorsed by the publisher.
